# Does the position of shoulder immobilization after reduced anterior glenohumeral dislocation affect coaptation of a Bankart lesion? An arthrographic comparison

**DOI:** 10.1007/s10195-015-0348-9

**Published:** 2015-04-17

**Authors:** Omid Reza Momenzadeh, Masoome Pourmokhtari, Sepideh Sefidbakht, Amir Reza Vosoughi

**Affiliations:** Bone and Joint Diseases Research Center, Department of Orthopedic Surgery, Chamran Hospital, Shiraz University of Medical Sciences, Shiraz, Iran; Department of Orthopedic Surgery, Jahrom University of Medical Sciences, Jahrom, Iran; Medical Imaging Research Center, Department of Radiology, Namazi Hospital, Shiraz University of Medical Sciences, Shiraz, Iran

**Keywords:** Shoulder, Dislocation, Bankart lesion, External rotation

## Abstract

**Background:**

The position of immobilization after anterior shoulder dislocation has been a controversial topic over the past decade. We compared the effect of post-reduction immobilization, whether external rotation or internal rotation, on coaptation of the torn labrum.

**Materials and methods:**

Twenty patients aged <40 years with primary anterior shoulder dislocation without associated fractures were randomized to post-reduction external rotation immobilization (nine patients) or internal rotation (11 patients). After 3 weeks, magnetic resonance arthrography was performed. Displacement, separation, and opening angle parameters were assessed and analyzed.

**Results:**

Separation (1.16 ± 1.11 vs 2.43 ± 1.17 mm), displacement (1.73 ± 1.64 vs 2.28 ± 1.36 mm), and opening angle (15.00 ± 15.84 vs 27.86 ± 14.74 °) in the externally rotated group were decreased in comparison to the internally rotated group. A statistically significant difference between groups was seen only for separation (*p* = 0.028); *p* values of displacement and opening angle were 0.354 and 0.099, respectively.

**Conclusion:**

External rotation immobilization after reduction of primary anterior shoulder dislocation could result in a decrease in anterior capsule detachment and labral reduction.

**Level of evidence:**

Level 2.

## Introduction

The most commonly dislocated joint in the human body is the glenohumeral joint [1]. Trauma is the main cause of primary anterior shoulder dislocation [2]. Recurrent dislocations and instabilities are the most common sequelae of primary anterior shoulder dislocation and are seen especially in young and active persons [3, 4]. A Bankart lesion or traumatic anterior detachment of the capsulolabrum complex is the principle pathology of further instabilities [5]. Treatment of anterior shoulder dislocation includes immobilization, immediate surgery or delayed surgery [4, 6].

Traditionally, to prevent recurrence of shoulder dislocation, the initial management of first-time anterior shoulder dislocation was immobilization in internal rotation after reduction followed by strengthening exercises of muscles around the shoulder joint. In recent years, multiple published articles reported better results after immobilizing the shoulder in external rotation [1, 7–12]. This prospective clinical trial was carried out to compare the effect of post-reduction shoulder immobilization positions, whether internal rotation or external rotation, on coaptation of the torn labrum.

## Materials and methods

**Fig. 1:**
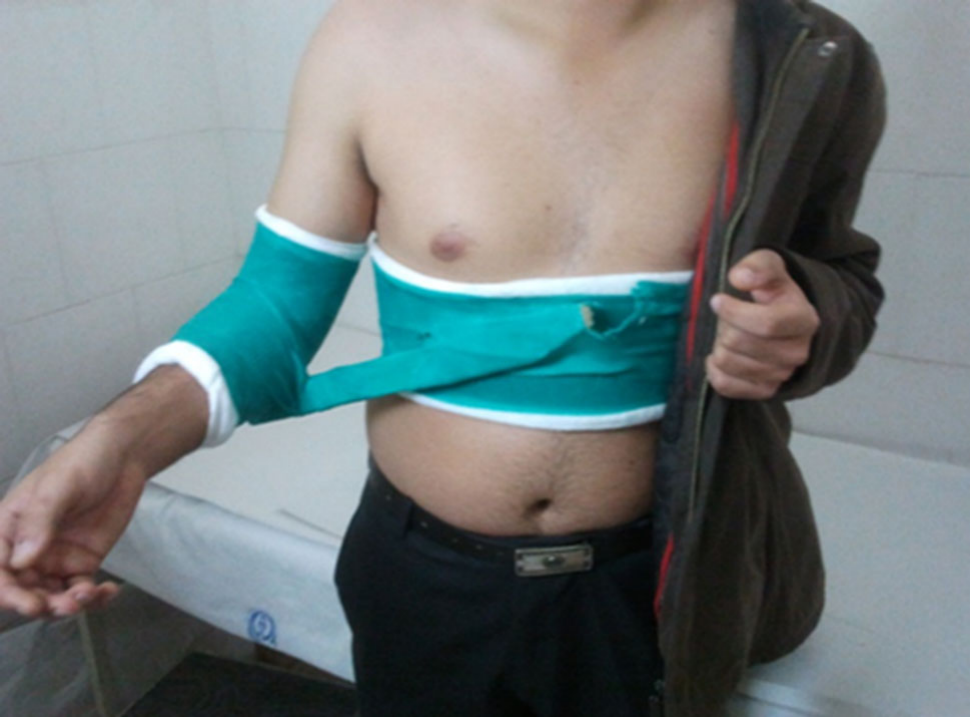
Shoulder spica cast to fix the arm in external rotation

Of the 60 patients with traumatic anterior shoulder dislocation from March 2012 to July 2012, only 35 cases were eligible to participate in this study. Exclusion criteria included associated fracture of the glenoid or the greater tuberosity approved by X-rays and computed tomography (CT), nerve damage, non-primary anterior dislocation, open reduction, and patients >40 years of age. Finally, 25 cases provided written informed consent before enrollment. After successful reduction, patients were randomized to either externally or internally rotated immobilization. All patients in the internal rotation group used a sling and swathe. Because of the high cost of a special external rotation brace, the arm was immobilized in a light comfortable shoulder spica cast with 10° external rotation (Fig. 1). Immobilization was continued for 3 weeks as performed in previous research [8, 11, 18]. Radiographic evaluation was then performed by an experienced radiologist blinded to the groups.

**Fig. 2:**
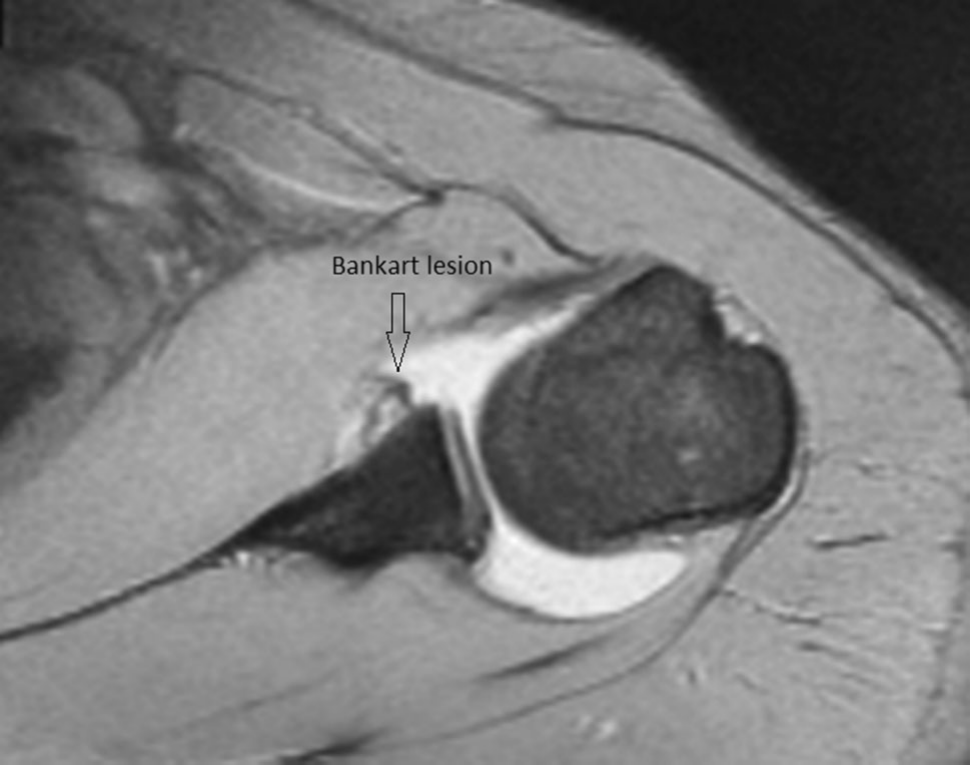
MRA of a 24-year-old male in the internal rotation group shows a Bankart lesion

Before injection, routine magnetic resonance imaging (MRI) sequences including T1 tse, T2 tse, and PD tse images in the oblique coronal plane and T2 tse and PD tse axial images as well as PD fat-saturated images in the oblique sagittal plane were obtained on a 1.5T GE scanner. A needle was then introduced into the glenohumeral joint through the rotator cuff interval [13]. A mixture of 10 cc omnipaque, 0.1 cc omniscan, 0.1 cc epinephrine, and 10 cc distilled water was injected under CT guidance. The patient was immediately taken to the MR scanner where T1 fat-saturated images in all three planes were obtained after immobilization of the arm using sandbags on the neutral/supinated hand. The slice thickness was 4 mm with a gap of 0.8 mm (Fig. 2).

**Fig. 3:**
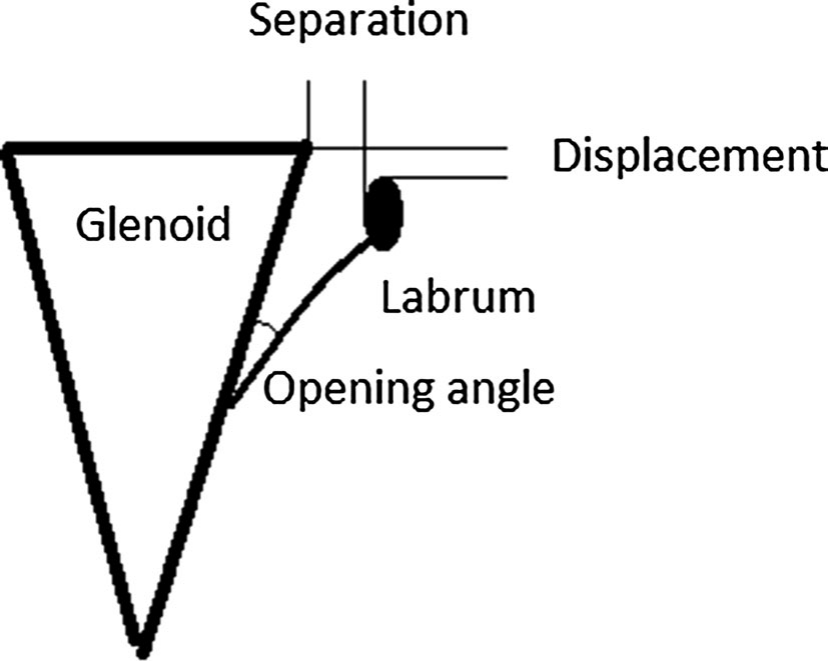
Schematic picture depicts radiographic parameters, i.e., displacement, separation, and opening angle

Parameters defined by Itoi et al. [7] were assessed in magnetic resonance arthrography (MRA). Separation is the distance in millimeters between the inner margin of the labrum and the anterior part of the glenoid neck. Displacement is defined as the distance in millimeters between the tip of the labrum and the tip of the glenoid rim. The opening angle is the angle between the articular aspect of the glenoid neck and a line tangential to the capsule at its glenoid insertion (Fig. 3). It is necessary to mention that separation and opening angle are not directly correlated because separation shows labrum translation at the level of the most lateral part of the glenoid but opening angle reveals the extent of capsular detachment from the glenoid.

SPSS version 18.0 for Windows was used for statistical analyses (SPSS Inc. Chicago, IL, USA). A Mann–Whitney test was performed.

## Results

Five patients were lost to follow-up. Hence, the externally rotated immobilization group consisted of nine patients and the internally rotated immobilization group consisted of eleven patients; all patients were male except one in the internally rotated group (Table 1).

**Table 1:** Demographic features and MRI parameters of all cases

Case number	Age (years)	Gender	Immobilization	Shoulder	Separation (mm)	Displacement (mm)	Angle (°)
1	31	M	External	Right	2.0	3.0	30
2	21	M	External	Right	2.5	5.0	43
3	19	M	External	Right	0	2.0	20
4	35	M	External	Left	0	0	0
5	32	M	External	Left	0	0	0
6	30	M	External	Right	2.0	2.0	22
7	18	M	External	Right	0	1.5	20
8	25	M	External	Left	2.0	2.1	0
9	34	M	External	Right	2.0	0	0
10	20	M	Internal	Right	3.1	2.0	57.5
11	40	M	Internal	Right	1.0	2.0	25
12	35	M	Internal	Right	0	0	17
13	22	M	Internal	Right	1.8	3.6	37
14	21	F	Internal	Right	3.4	4.0	30
15	25	M	Internal	Right	4.0	3.2	21
16	33	M	Internal	Right	3.2	1.8	0
17	28	M	Internal	Left	2.0	2.0	18
18	18	M	Internal	Right	2.2	0	30
19	19	M	Internal	Right	3.0	3.5	31
20	22	M	Internal	Right	3.1	3.0	40

*mm* millimeters, *M* male, *F* female

Comparison of the imaging parameters of the two groups, as shown in Table 2, shows all variables decreased in the externally rotated immobilization group; therefore, the labrum coaptated in a near anatomical position when the arm immobilized in external rotation. The labrum of two patients in the externally rotated group (20 % of cases) had been perfectly located in its original position with a zero measurement for displacement, separation, and open angle.

**Table 2:** Description of age, radiographic parameters, and *p* values

	Mean age (years)	Separation (mean ± SD)	Displacement (mean ± SD)	Open angle (mean ± SD)
Externally rotated immobilization group	27.2	1.16 ± 1.11 mm	1.73 ± 1.64 mm	15.00 ± 15.84°
Internally rotated immobilization group	25.7	2.43 ± 1.17 mm	2.28 ± 1.35 mm	27.86 ± 14.74°
*p* value		0.028	0.354	0.099

*SD* standard deviation, *mm* millimeters

## Discussion

Traditionally, reduced anterior shoulder dislocation was immobilized in adduction and internal rotation and reduced posterior dislocation was immobilized in abduction and external rotation [14]. Approximately 15 years ago, Itoi et al. [15] defined the coaptation zone of a Bankart lesion to the glenoid in a cadaveric study. Itoi et al. [7] then evaluated coaptation of the torn labrum in internal rotation and external rotation using MRI. They concluded that external rotation immobilization approximates the Bankart lesion more than internal rotation. Moreover, another study by Itoi et al. [1] reported a decrease in recurrence rate of anterior shoulder dislocation at a mean follow-up of 15.5 months in patients with the arm immobilized in external rotation after glenohumeral reduction. Other studies in cadavers [16] and in humans using MRI [9, 12] and arthroscopy [10] showed coaptation of the labrum and increase of the labrum−glenoid contact force after immobilization in external rotation. This position improves approximation of the Bankart lesion by placing greater tension on the subscapularis, anterior capsule, and ligaments, closing the anterior joint cavity, and bringing the labrum back to the glenoid rim [1, 7].

Clinically, satisfactory results with regard to instabilities and recurrence rates of dislocation (0.0–19.0 %) have been reported [1, 8, 11, 17, 18]. Although patients with primary anterior shoulder dislocation immobilization in external rotation may have more benefits than in internal rotation, some reported contradictions and controversies should be mentioned.

1. The optimum position of immobilization in external rotation and its duration has not been clearly determined [4, 12, 19].
2. Multiple studies reported conflicting results on acceptance of external rotation braces by patients [1, 20].
3. External rotation immobilization after first-time anterior shoulder dislocation has not been well accepted by orthopedic surgeons, e.g., approximately 93 % of orthopedic surgeon in England preferred internal rotation immobilization after reduction of anterior shoulder dislocation [21].
4. Recent multiple clinical trials have not supported the effectiveness of immobilization in external rotation compared with internal rotation to prevent further instabilities [22–26].

In our study, separation decreased to a larger extent in the externally rotated immobilization group than in the internally rotated group (1.16 ± 1.11 vs 2.43 ± 1.17 mm; *p* = 0.028); the *p* value of displacement and opening angle showed no statistically significant difference. Our results are the same as those reported by Liavaag et al. [9].

The main limitation of this study is the small number of cases in each group. Moreover, the review of MRA by only one radiologist, the lack of clinical confirmation of stability of the joint especially in the long-term follow-up period, and the absence of questioning patient satisfaction are other limitations.

We would suggest external rotation immobilization after reduction of primary anterior shoulder dislocation for decreasing anterior capsule detachment and labral reduction. Long-term clinical trials may be required to confirm its clinical usage.
